# GVES: machine learning model for identification of prognostic genes with a small dataset

**DOI:** 10.1038/s41598-020-79889-5

**Published:** 2021-01-11

**Authors:** Soohyun Ko, Jonghwan Choi, Jaegyoon Ahn

**Affiliations:** 1 Department of Computer Science and Engineering, Incheon National University, Incheon, Republic of Korea; 2 Department of Computer Science, Yonsei University, Seoul, Republic of Korea

**Keywords:** Cancer, Computational biology and bioinformatics

## Abstract

Machine learning may be a powerful approach to more accurate identification of genes that may serve as prognosticators of cancer outcomes using various types of omics data. However, to date, machine learning approaches have shown limited prediction accuracy for cancer outcomes, primarily owing to small sample numbers and relatively large number of features. In this paper, we provide a description of GVES (Gene Vector for Each Sample), a proposed machine learning model that can be efficiently leveraged even with a small sample size, to increase the accuracy of identification of genes with prognostic value. GVES, an adaptation of the continuous bag of words (CBOW) model, generates vector representations of all genes for all samples by leveraging gene expression and biological network data. GVES clusters samples using their gene vectors, and identifies genes that divide samples into good and poor outcome groups for the prediction of cancer outcomes. Because GVES generates gene vectors for each sample, the sample size effect is reduced. We applied GVES to six cancer types and demonstrated that GVES outperformed existing machine learning methods, particularly for cancer datasets with a small number of samples. Moreover, the genes identified as prognosticators were shown to reside within a number of significant prognostic genetic pathways associated with pancreatic cancer.

## Introduction

The accurate identification of genes with prognostic value in the prediction of cancer outcomes is a challenging task for cancer researchers. Numerous statistical and computational methods have been developed to increase the accuracy of cancer prognosis^1^. Also, relatively recently, machine learning techniques have been applied to various omics datasets (e.g., gene expression), to identify genes capable of serving as prognosticators of cancer outcomes^2–5^.

Although machine learning techniques are powerful, they are associated with a fundamental challenge, namely that the number of dimensions (e.g., individual genes or genetic loci) is relatively very large in comparison to the number of samples^6^. Reducing a genetic dimension using feature selection or through incorporation of additional biological network data such as protein–protein interaction (PPI) may assist in overcoming this challenge. As the genes in a prognostic gene module can be treated as relevant features for classification or regression methods, these approaches may be associated with improved prediction accuracies compared with traditional statistical methods. An additional strength of such approaches is that the identified prognostic gene modules can provide insights into the biological processes or functions associated with tumor progression. Prognostic gene modules can be identified using computational methods including network clustering algorithm^7^ or Google’s PageRank^3,5^. Recently, machine learning algorithms such as Word2Vec^2^ or GANs^4^ have been applied to biological networks to identify prognostic gene modules with improved performance.

Although previously described methods work well for cancer datasets with relatively large sample numbers (e.g., breast cancer dataset), their prediction accuracy can be significantly limited for cancer datasets with small sample numbers (e.g., pancreatic cancer dataset). Moreover, deep learning techniques are prone to overfitting when sample sizes are small.

Nonetheless, if the challenges associated with small sample sizes can be overcome, deep learning techniques could be efficiently used for more accurate identification of genes with prognostic value and the prediction of cancer prognoses. Our previous work^2,4^ showed that deep generative models such as Word2Vec^8^ or GANs^9^ can be effectively used to detect prognostic gene modules. Graph neural network^10^(GNN) is another promising technique to achieve the same purpose. GEDFN^11^ used GNN to predict disease outcome by integrating gene network information.

In this paper, we describe the proposed GVES (Gene Vector for Each Sample). GVES is composed of three steps. First, genes are scored using the t-test for each sample, to construct an FI (Functional Interaction)^12^ network containing genes scored for each sample. Second, for each sample, the random walk algorithm is performed on the scored FI network multiple times, to produce sequences of genes. We refer to a sequence of genes as a gene path. If genes are envisioned as words, a gene path can be a sentence. As the CBOW model can predict a target word by examining preceding and following words in the sentence in natural language processing, CBOW can also predict a target gene by examining its neighboring genes in gene paths. If CBOW is trained to predict target words effectively, embedding vectors of words can be obtained. Likewise, genes are also represented by embedding vectors, referred to as gene vectors. So, we can get gene vectors for all genes, for each sample after step two. Third, samples are clustered to reduce heterogeneity to form groups and re-clustered within each group using their gene vectors to calculate normalized mutual information for each gene. Because the number of gene paths is solely dependent on the number of random walking, a sufficient number of gene paths can be obtained to train the CBOW model well, thereby attaining accurate gene vectors. Also, since generation of gene paths is not dependent on the number of samples, GVES can be effective on small sample data.

The proposed method was applied to the gene expression data of six cancer types: Breast invasive carcinoma (BRCA), kidney renal clear cell carcinoma (KIRC), liver hepatocellular carcinoma (LIHC), lung adenocarcinoma (LUAD), pancreatic adenocarcinoma (PAAD) and stomach adenocarcinoma (STAD). The proposed method outperforms existing methods, especially for datasets with small sample sizes. Importantly, genes identified as prognosticators were enriched in many PAAD-related biological functions or pathways, allowing the suggestion of novel prognostic genes and their role in known functions or pathways.

## Results

### Overview of the proposed model

**Figure 1:**
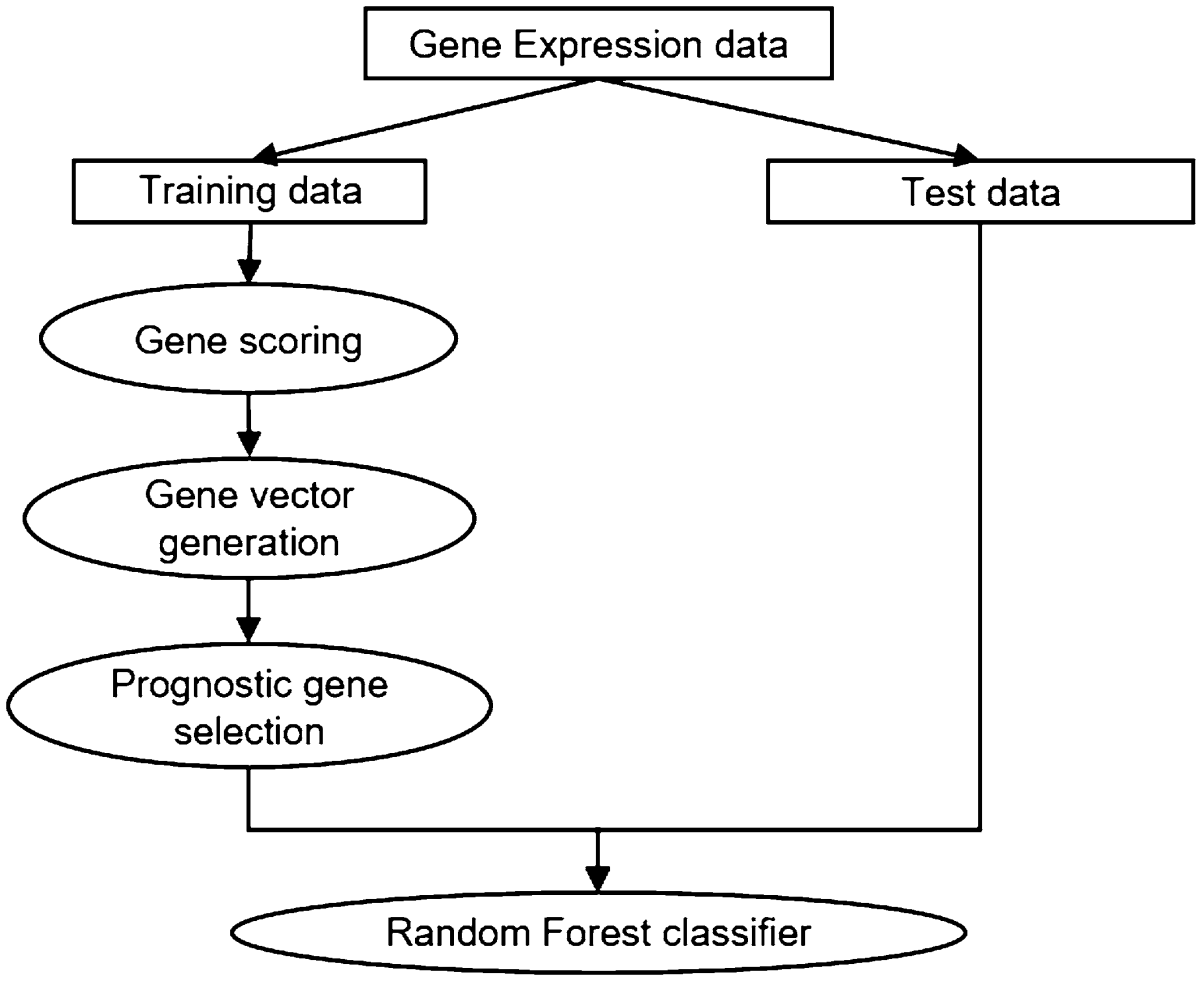
Overview of proposed model. The proposed model has three steps: (1) measuring gene scores; (2) generating gene vectors; and (3) extracting prognostic genes.

The proposed model consists of three steps, as shown in Fig. 1. First, gene scores for each sample are calculated and genes are selected. Second, gene vectors for genes selected in first step are generated for each sample using CBOW. Lastly, genes are selected using gene vectors and used to predict cancer outcomes using random forest. Each step is described in detail in the Methods section.

### Description of data

We used the TCGA-Assembler^13^ to collect mRNA and clinical data for six cancer types from the Cancer Genome Atlas (TCGA)^14^. The six types of cancer included breast cancer (BRCA), kidney cancer (KIRC), liver cancer (LIHC), lung cancer (LUAD), pancreatic cancer (PAAD), and stomach cancer (STAD). The clinical data included information about the survival status and survival duration of patients with cancer. Patients were assigned to a "poor" prognosis group if their death falls within a criterion in Table 1, and assigned to a "good" prognosis group if they survived longer than a criterion in the same table.

**Table 1:** Descriptions of mRNA data for each cancer type.

Cancer type	#Good prognosis	#Poor prognosis	Criterion for label	#Genes
BRCA	91	63	5 years	11,577
PAAD	20	24	1 years	11,403
STAD	29	16	1 years	11,570
LIHC	77	57	1 years	11,439
LUAD	52	53	2 years	11,472
KIRC	65	47	4 years	11,569

We also used the FI network as a biological network, which was downloaded from the Reactome database^12^. The FI network is composed of protein–protein interactions (PPIs), gene coexpression, protein domain interaction, gene ontology (GO) annotations, and text mined protein interactions. From each mRNA dataset, we removed genes not included in the FI network. Information relating to the mRNA data used is presented in Table 1.

### Identification of optimal hyper-parameters

**Figure 2:**
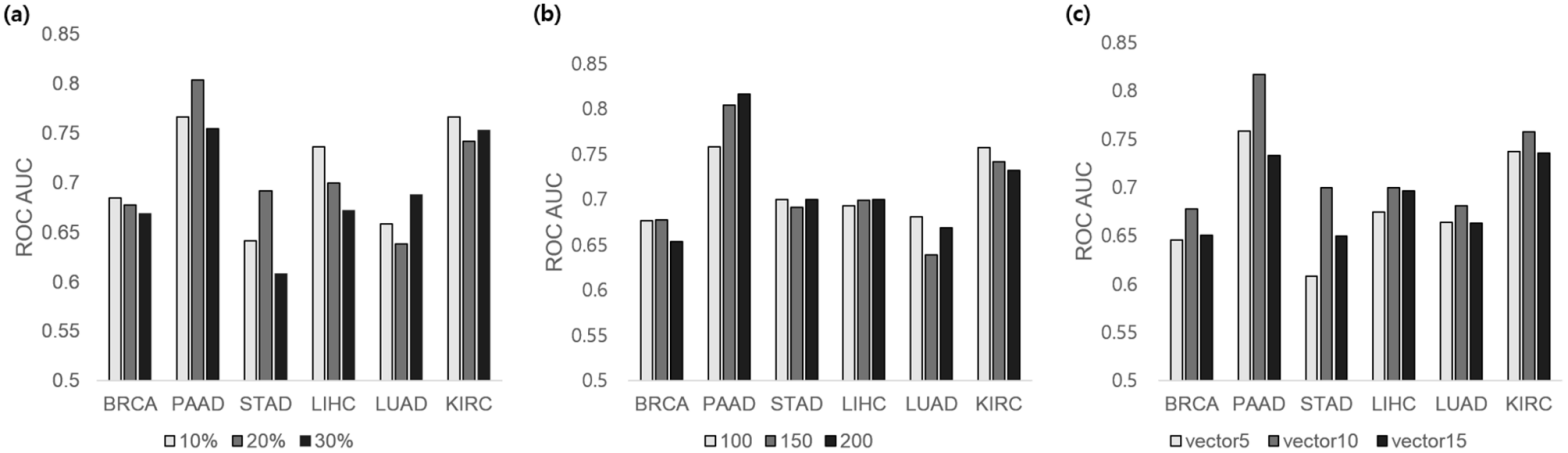
The fivefold cross validation results for finding optimal parameters. **(a)** Ratio of nodes selected for reconstruction of FI network (*r*), **(b)** number of genes to select (*n*), **(c)** size of gene vector (*v*).

We performed fivefold cross validation to identify optimal parameters including the ratio of nodes selected for reconstruction of the FI network (*r*), the number of genes to select (*n*), and the size of the gene vector (*v*). Figure 2a,b reveal that the optimal *r* and *n* vary according to cancer type, but the differences are subtle. Therefore, we selected the *r* and *n* values associated with the best average area under the curve(AUC), 20% and 150, respectively. The size of the gene vector, *v*, was set as 10, the value associated with the best AUC for all cancer types, as shown in Fig. 2c. The hyper-parameters used are presented in Table 2.

**Table 2:** Optional parameters of each cancer type.

Cancer type	*r*	*n*	*v*	*K*	#Estimator of random forest
BRCA	20	150	10	4	50
PAAD				3	50
STAD				2	30
LIHC				2	50
LUAD				3	50
KIRC				2	30

**Figure 3:**
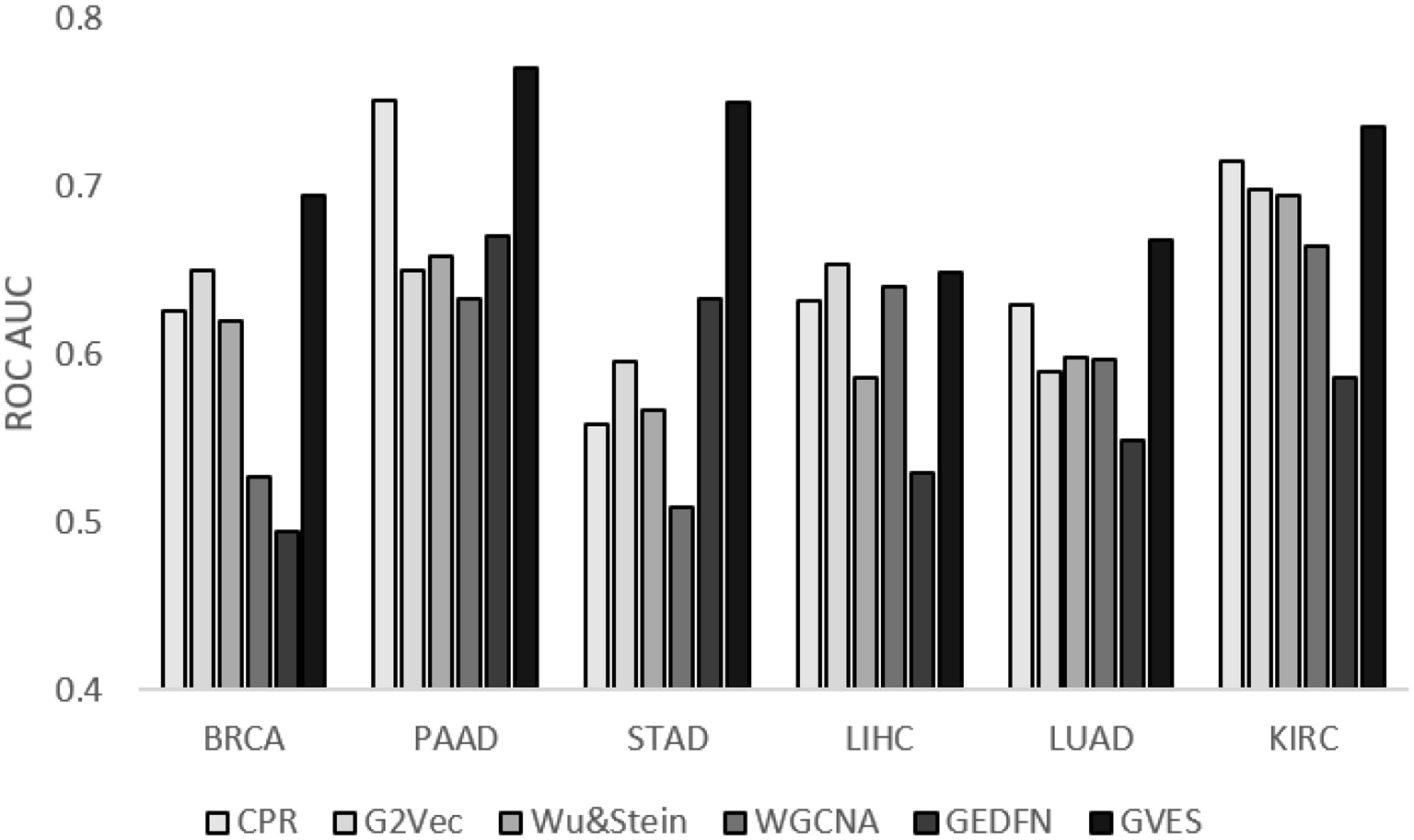
The tenfold cross validation results for each cancer type. Y-axis indicates mean AUC of tenfold cross validations for each cancer type.

We measured AUC values of GVES using tenfold cross validation and optimal hyper-parameters for each cancer type, and compared these to the AUC values of existing methods including CPR^3^, G2Vec^2^, Wu & Stein^15^, WGCNA^7^ and GEDFN^11^. The optimal hyper-parameters for those methods are provided in Supplementary Table 1. GVES outperformed those methods, especially for PAAD and STAD, which have relatively small sample sizes, as shown in Fig. 3 and Supplementary Table 4.

### Prognosis prediction using small number of samples

**Figure 4:**
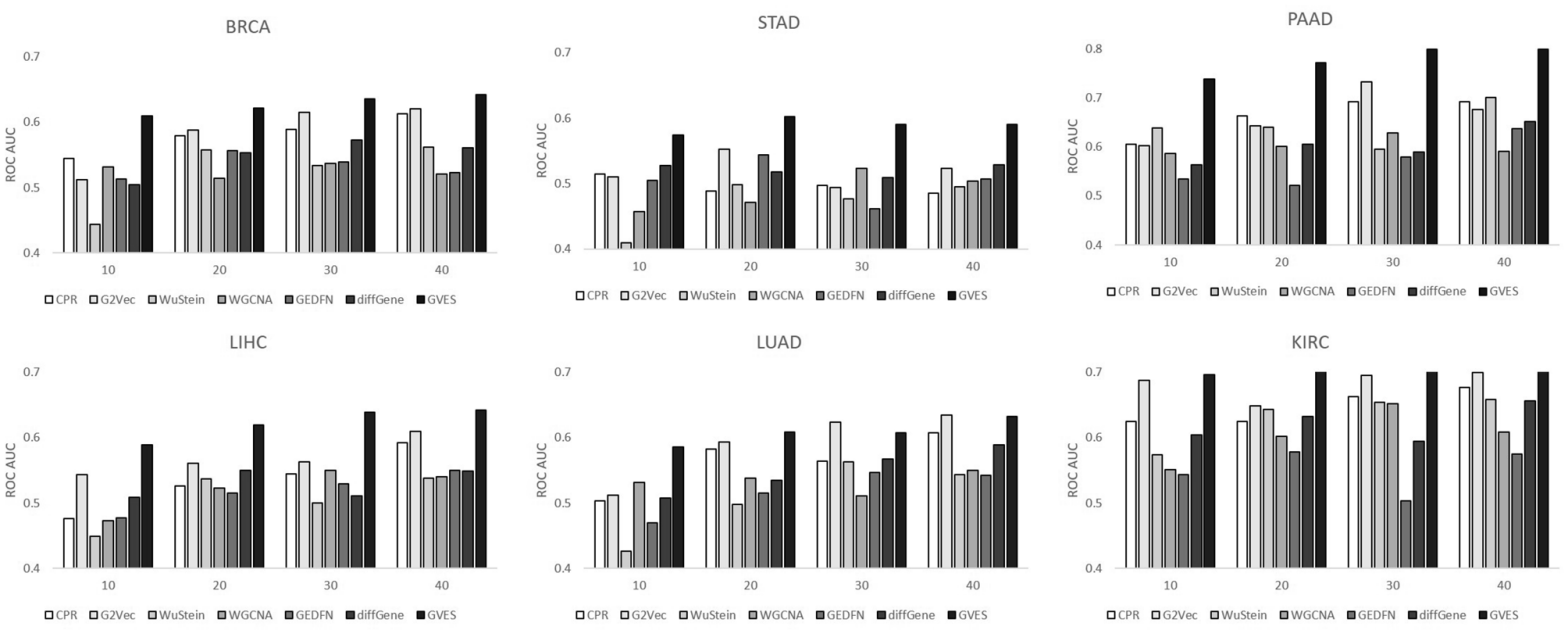
AUC measured for each cancer type varying sample size. Y-axis indicates mean AUC of fivefold cross validations for each cancer type varying number of samples used for training.

To characterize the effect of sample size in more detail, we generated five sets of training data using 10, 20, 30, and 40 randomly selected samples for each cancer type. The number of randomly selected good and poor prognosis samples was identical for each training dataset. For each training dataset, samples that were not selected were used as a test dataset. The AUC values in Fig. 4 are the average from five datasets. To provide evidence with a higher confidence level for sample size effect of GVES, we additionally selected differentially expressed genes by fold change and p-value and fed them into random forest (*diffGene* in Fig. 4). The thresholds for fold change and p-value are 1.5 and 0.05, respectively. Genes are sorted in descending order to fold change, and the top *n* genes are selected, where *n* is the number of genes that GVES selected. If the number of genes after thresholding is less than *n*, all genes after thresholding are selected.

We note that, unlike comparator methods, GVES showed no significant differences in AUCs for different sample sizes. We can also confirm that GVES outperformed these comparator methods in all cases except those using 30 and 40 samples of the LUAD dataset.

### Functional analysis of the gene module

**Figure 5:**
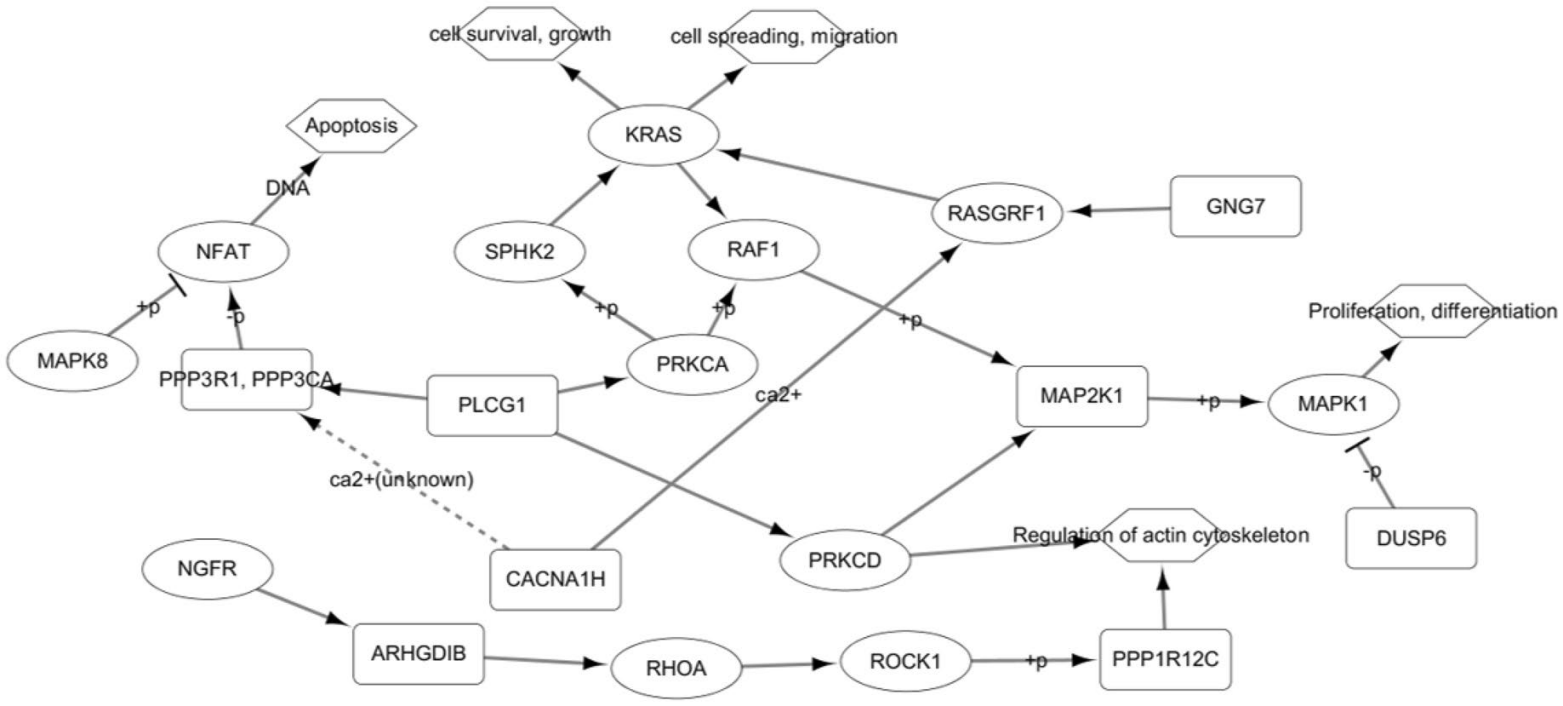
Part of PAAD-related genetic pathway drawn using enriched KEGG pathways. Rectangular node indicates genes identified by GVES.

We selected the top 150 scored genes using whole gene expression data of PAAD, and performed functional annotation analysis using DAVID^16,17^. The top 150 genes for all cancer types are provided in Supplementary Table 2. We were able to identify numerous GO terms and pathways related to PAAD. The complete functional analysis results are provided in Supplementary Table 3. We selected some interesting KEGG pathway^18^ and visualized them using Cytoscape^19^ in Fig. 5.

**Figure 6:**
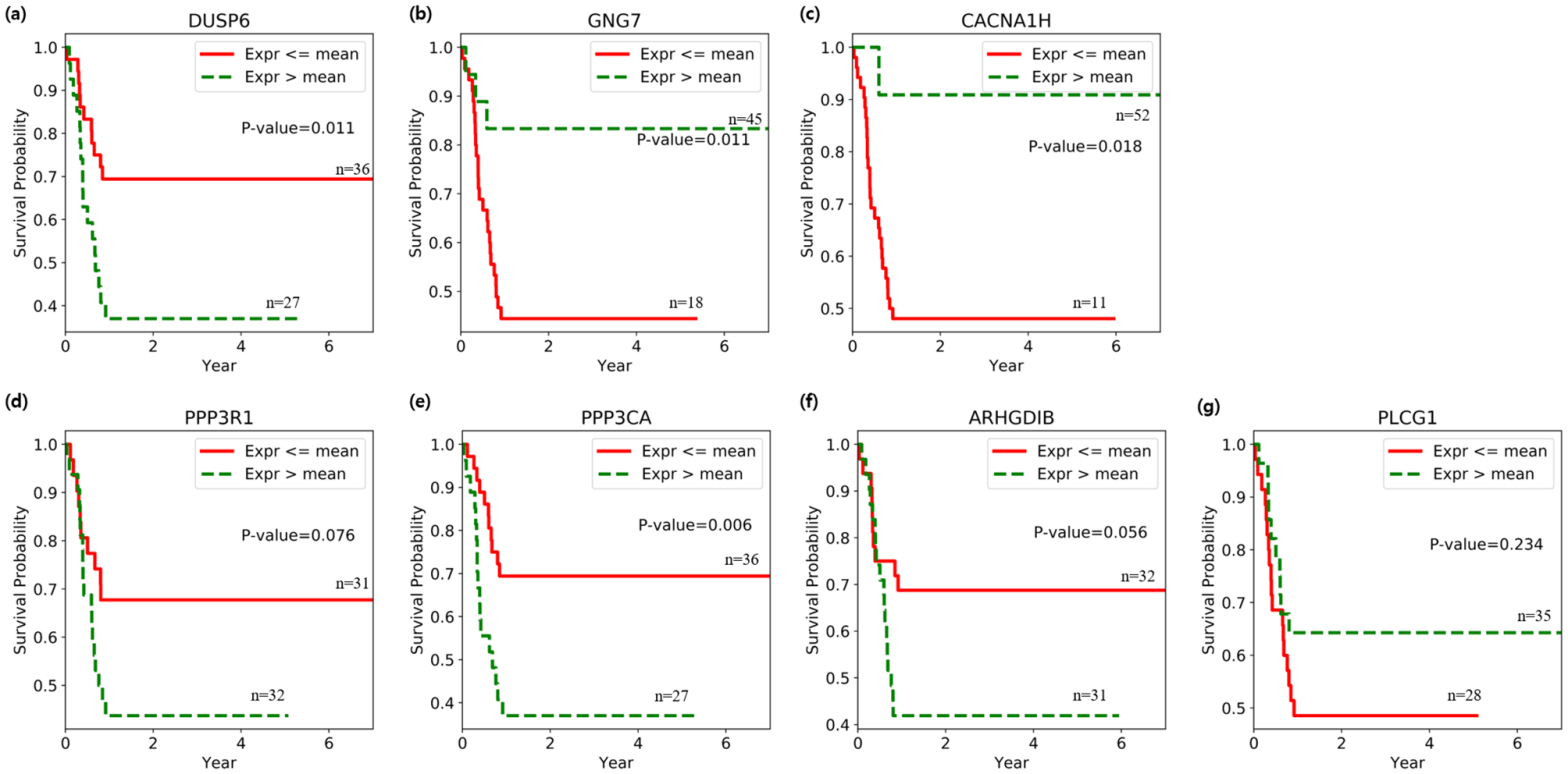
Kaplan–Meier curves. Red line refers to survival probability of patients providing samples for which gene expression values are greater than or equal to average, and green line means survival probability of samples with expression values less than average. **(a)** *DUSP6*, **(b)** *GNG7*, **(c)** *CACNA1H*, **(d)** *PPP3R1*, **(e)** *PPP3CA*, **(f)** *ARHGDIB*, **(g)** *PLCG1*.

Mutations within *KRAS* and *BRAF*, and histone deacetylation of *DUSP6* synergistically contribute to the activation of MAPK, which activates a number of genes that may be related to the malignant phenotypes of pancreatic cancer^20,21^. It has been shown that exogenous overexpression of *DUSP6* induces the inactivation of MAPK1 when endogenous expression of *DUSP6* is low^22^. Figure 6a shows that endogenous expression of *DUSP6* is high in the poor outcome group (p-value = 0.011), indicating that a therapy designed to activate *DUSP6* may not work in this group.

*KRAS* is known to be a driver gene for pancreatic cancer^23^. As shown in Fig. 5, within the KEGG pathway, *KRAS* is affected by *RASGRF1*. It has been studied that overexpression of *RASGRF1* can inhibit cell proliferation and cell invasion in colorectal cancer^24^. We also note the impact of *GNG7* and *CACNA1H* on *RASGRF1*. Since *GNG7* regulates *RASGRF1*, we suspect that low expression of *RASGRF1* by *GNG7* can lead to an increase in cell proliferation and cell invasion, also in pancreatic cancer. In fact, as illustrated in Fig. 6b, the expression of *GNG7* is low in the poor outcome group (p-value = 0.011). Figure 6c shows that low expression of *CACNA1H* is also associated with poor outcomes (p-value = 0.018). We can hypothesize that reduced expression of *CACNA1H* prevents Ca^2+^ influx for *RASGRF1*, which contributes to poor outcomes in patients with pancreatic cancer.

In Fig. 5, we can see that *CACNA1H* also affects *PPP3R1* and *PPP3CA*, genes that encode calcineurin, by an unknown mechanism, as well as *RASGRF1*. We observed *PPP3CA* is significantly overexpressed (p-value = 0.006) and *PPP3R1* is weakly overexpressed (p-value = 0.076) in the poor outcome group in Fig. 6d,e, which supports findings from an existing study noting that the dephosphorylation of NFAT by calcineurin is transported to the nucleus and regulates numerous genes essential for various biological functions, as well as the development and metastasis of pancreatic cancer^25^.

We also showed that overexpression of *ARHGDIB* is weakly associated with poor outcomes (p-value = 0.056) as shown in Fig. 6f. *ARHGDIB* is significantly up-regulated in pancreatic cancer cell^26^. We suspect that *ARHGDIB* may affect the structure of the actin cytoskeleton, and eventually cell motility and metastasis^27,28^.

One of the most interesting genes identified using the approach described here is *PLCG1*, a hub gene that connects many pathways related to pancreatic cancer outcomes. It is thought that *PLCG1* affects the structure of the actin cytoskeleton like *ARHGDIB* or the calcineurin like *CACNA1H*, or that it may be an upstream gene of the RAS and MAPK signaling pathway. *PLCG1* has also been shown to be involved in colorectal tumorigenesis by means of crosstalk with STAT3^29^; however, its role in pancreatic cancer remains unknown, to the best of our knowledge. We predict that *PLCG1* is an upstream gene of many pancreatic cancer-related pathways, and may sensitively control those pathways. Figure 6g, illustrates that that poor outcome group shows weak under-expression of *PLCG1* (p-value = 0.234)*.*

## Discussion

In this study, we describe the proposed GVES, developed with the goal of more accurately identifying genes with prognostic value even in cases of small sample sizes of datasets. GVES is based on the Word2Vec model and generates vector representations of genes using gene expression and biological network data. Prognostic genes identified by GVES are those in which gene vectors are distinctive for good and poor outcome patient groups.

The fundamental concept of GVES is that it generates gene vectors for each sample, thereby limiting the effect of sample size. We report that GVES outperformed existing machine learning methods for all cancer types, especially in cases of small sample sizes, and prediction accuracies were not significantly decreased even when the number of good and poor samples was as low as 10 for six cancer types. We also performed a functional analysis on the genes identified as potential prognosticators using pancreatic cancer as the model, and confirmed that many were associated with GO terms and pathways as supported by numerous existing studies.

GVES can be useful for data with small sample sizes. However, since a gene vector generation step is performed for each sample, running time can be long, a fundamental disadvantage of GVES. Another disadvantage of GVES is that it has many hyper-parameters (Table 2) that must be optimized. We are planning an upgraded version of GVES, which requires fewer hyper-parameters.

## Methods

### Gene scoring

**Figure 7:**
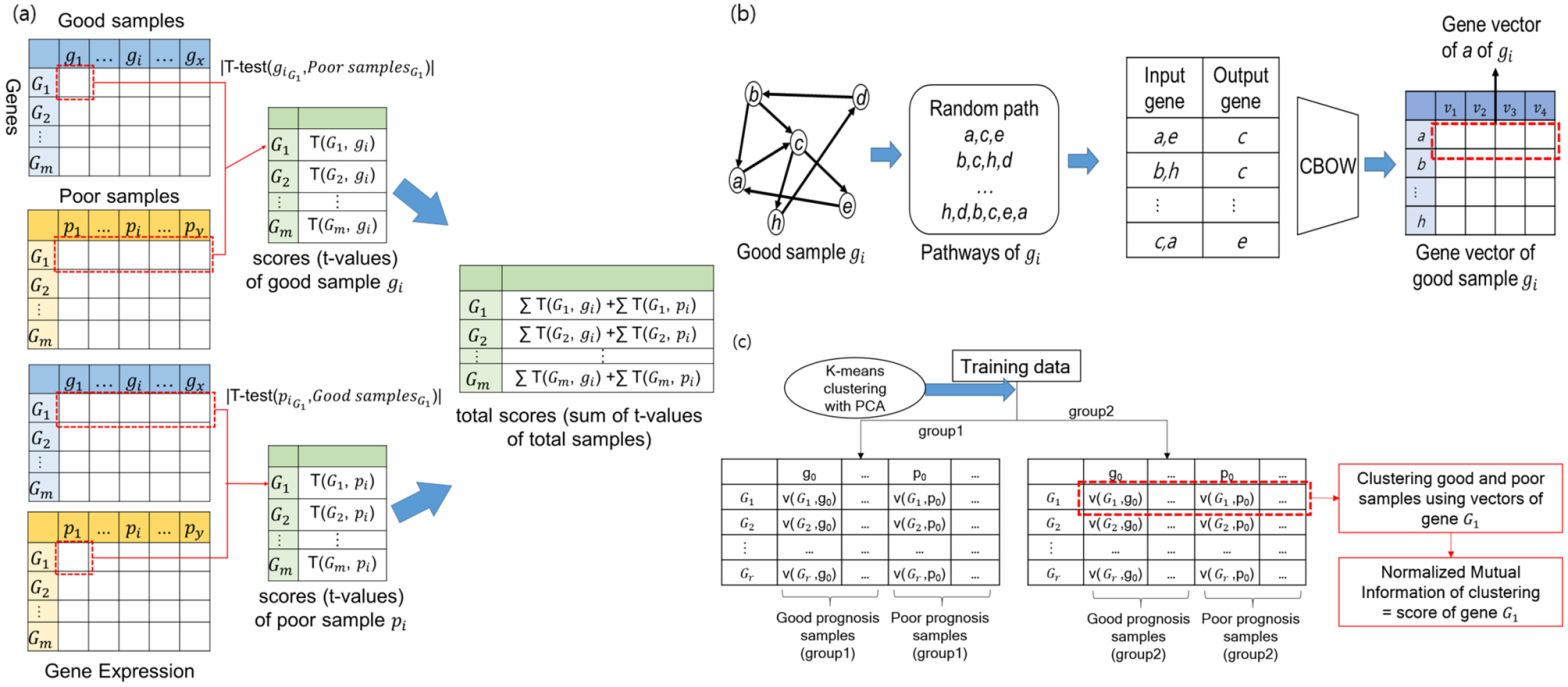
Detailed procedure for each step **(a)** gene scores are calculated using t-test for each sample, **(b)** for each sample, gene paths are generated through random walk on FI network of which genes are scored. Generated gene paths are fed into CBOW model to obtain gene vectors, **(c)** samples are clustered to reduce heterogeneity to form groups by *k*-means and PCA, and then re-clustered within each group using their gene vectors to calculate normalized mutual information for each gene.

First, we calculate scores of genes. For each gene, ${G}_{i}$, a t-value is calculated using a one-sample t-test for the expression value of ${G}_{i}$ of each sample in the good outcome group and those of all samples in the poor outcome group. Likewise, a t-value for each sample in the poor outcome group is calculated for all samples in the good outcome group. All t-values are summed to generate total scores of gene ${G}_{i}$. Genes with higher total scores are likely to show higher differences in expression values between good and poor outcome groups. We select genes with the top *r*% of total score. This process is illustrated in Fig. 7a.

**Table 3:** Descriptions of hyper-parameters.

Hyper parameter	Description
*r*	The ration of node selected for reconstruction of FI network
*n*	The size of gene vector
*v*	The number of prognostic genes
*k*	Used for *k*-means clustering to reduce sample heterogeneity

### Gene vector generation

Next, we generate a gene vector for the top *r*% genes that were selected in the gene scoring process. A gene vector is generated using CBOW, a word embedding model that maps words of sentences into vectors. Here, we can think of a word and sentences as a gene and gene paths, respectively. To generate gene paths, random walk is applied to the gene network numerous times. The gene network used in this study is the FI network^12^, but reconstructed with only the top *r*% genes.

Each gene in this network has t-values for each sample from the good and poor groups, calculated in the gene scoring process. We generate gene paths by applying random walk to the network for each sample. There are three constraints when applying random walk: (1) visited nodes are not visited again; (2) a probability of moving to a gene is proportional to the t-score of that gene; and (3) there is a maximum path length, which is set as 80. Random walk is performed ten times for each gene node as a starting node. For example, if the number of genes is 1000, we can obtain gene 10,000 gene paths for each sample.

CBOW is trained using input genes and an outcome gene for each gene path. For each gene in a gene path as an outcome gene, input genes comprise those within a window of size 1 of an outcome gene. A neural network is trained to accurately predict an outcome gene given input genes. A gene vector represents gene specific information in the context of a gene network for a given sample.

As the described process is applied to each sample, each sample has its own gene vector of size *v*. This process is illustrated in Fig. 7b.

### Prognostic gene selection and prognosis prediction

In this section, we select genes for outcome prediction using the gene vectors obtained in the gene vector generation process. The first step in selecting a gene is clustering heterogeneous cancer samples. In our previous study, we demonstrated that heterogeneous biomarker genes by sample clustering aids in the classification of cancer outcomes^3^. Similarly, we used principal component analysis (PCA) and *k*-means clustering to divide the entire sample into *k* sample groups with similar gene expression patterns. Gene expression data are then reduced to two dimensions by PCA, and *k*-means clustering is applied to the reduced data. The optimal *k* is obtained using a silhouette coefficient, and *k* sample groups are obtained.

For each sample group, we again divide the samples into two subgroups of samples, for each gene ${G}_{i}$, using *k*-means clustering with *k* = 2. A distance between two samples is the same as a distance between their gene vectors of ${G}_{i}$ for a given gene. After samples are clustered, a score of ${G}_{i}$ is calculated using Normalized Mutual Information (NMI), as follows:

1 $$Score\left({G}_{i}\right)=NMI(RL,CL)=\frac{2 \times MI(RL;CL)}{[H\left(RL\right)+H(CL)]}$$

where *RL* and *CL* are vectors of real and predicted labels of samples, respectively, and *MI* and *H* refer to mutual information and entropy, respectively. A score of a gene implicates its purity of sample labels.

This process is illustrated in Fig. 7c. Consequently, we can calculate scores for *k* sample groups, for each gene. The score of a gene is the sum of *k* scores. Genes with higher scores would accurately divide the two sample groups into good and poor outcome groups. We select the top *n*-scored genes and use them for classification through random forest^30^. We summarize the hyper-parameters used throughout this process in Table 3.

## Supplementary Information

Supplementary information.
